# Early Postural EMG Dynamics During Optic Flow: Effects of Time, Velocity, Retinal Location and Laterality

**DOI:** 10.3390/brainsci16101090

**Published:** 2026-10-10

**Authors:** Milena Raffi, Aurelio Trofè, Alessandra Laffi, Andrea Meoni, Alessandro Piras

**Affiliations:** 1 Department of Biomedical and Neuromotor Sciences, University of Bologna, 40126 Bologna, Italy; alessandra.laffi3@unibo.it (A.L.); andrea.meoni@unibo.it (A.M.); 2 Department of Quality of Life, University of Bologna, 47921 Rimini, Italy; aurelio.trofe2@unibo.it (A.T.); alessandro.piras3@unibo.it (A.P.)

**Keywords:** optic flow, postural control, electromyography, early motor response, stimulus velocity, retinal stimulation, paraspinal muscles, tibialis anterior, sensorimotor integration, time-domain analysis

## Abstract

**Highlights:**

**What are the main findings?**

- EMG activity increased from the 0–200 ms to the 200–400 ms window after optic-flow onset in all four muscles.
- The influence of optic-flow velocity, retinal location and laterality differed across postural muscles.

**What are the implications of the main findings?**

- Time-resolved bilateral EMG is a useful complementary approach to whole-trial averaging of postural responses.
- Bilateral recordings are important because averaging left and right muscles may conceal lateralised responses.

**Abstract:**

**Background/Objectives:** Optic-flow velocity is known to influence postural sway, whereas corresponding electromyographic (EMG) effects may be difficult to detect when muscle activity is averaged over long stimulation periods. We therefore investigated the early temporal organisation of muscle activity following optic-flow onset and examined whether this response was modulated by stimulus velocity, retinal stimulation location, and laterality. **Methods:** Surface EMG recordings from the tibialis anterior (TA), soleus (SOL), trapezius descendens (TD), and paraspinal C4 muscles were reanalysed from a previously published dataset of 18 healthy adults exposed to radial optic flow at five velocities and three retinal stimulation configurations. RMS activity was quantified in two consecutive post-stimulus windows, 0–200 ms and 200–400 ms, and analysed using repeated-measures ANCOVA with baseline activity entered as a covariate. **Results:** All four muscles showed greater activation during the 200–400 ms window than during the 0–200 ms window, indicating a consistent temporal increase in EMG activity shortly after optic-flow onset. Additional effects were muscle-specific. In TA, the temporal increase was greater during large-field than peripheral stimulation. In SOL, temporal modulation varied with velocity and body side. TD showed a significant effect of velocity. C4 activity was greater on the right than on the left side. **Conclusions:** Early postural muscle activity shows a common temporal increase during the first 400 ms after optic-flow onset, together with significant stimulus modulation and laterality. These findings suggest that time-resolved bilateral EMG can reveal aspects of the early postural response that may be hidden when muscle activity is averaged over longer periods.

## 1. Introduction

When moving through the environment, the visual system receives a continuous motion pattern known as optic flow, which provides essential information for the perception of self-motion, heading direction and postural orientation [1,2]. The neural processing of optic flow involves a distributed cortical and subcortical network, which integrates visual motion signals with proprioceptive and vestibular inputs to generate coordinated postural responses [3,4,5,6,7,8].

The relationship between optic flow and postural control has been extensively investigated using stabilometric and electromyographic approaches [9,10,11]. Several studies have demonstrated that the spatial properties of optic flow, including retinal stimulation location, expansion pattern and speed, modulate the magnitude and direction of postural responses [12,13,14,15]. Central and peripheral vision differ in spatial resolution, motion sensitivity and their relative contribution to visually guided behaviour. Peripheral visual stimulation has often been associated with visually evoked postural responses, whereas central vision is generally more important for high-acuity spatial analysis [2,15,16,17,18,19].

The speed of optic-flow stimuli represents a functionally relevant parameter for postural control, as it encodes the velocity of self-motion through the environment and directly influences heading perception and trajectory planning [20,21]. However, a consistent finding using long trial durations is the absence of significant speed-dependent modulation of postural muscle activity when analysed over the entire stimulus period [14]. This apparent discrepancy between postural sway effects and muscle activity has been attributed to motor adaptation mechanisms that develop progressively during sustained visual stimulation, effectively masking the phasic neuromuscular responses elicited at stimulus onset [14].

This interpretation is supported by convergent evidence from the motor learning literature. Dionne and Henriques [22] demonstrated that human subjects progressively learn to correct visual perturbations over successive trials, and several studies have shown that the nervous system generates compensatory responses that mirror the perturbation, reducing its visible effects on motor output over time [23,24]. Applied to postural control under optic-flow stimulation, these findings suggest that mean muscle activity computed over 30 s trials may substantially underestimate the true magnitude of the initial neuromuscular response, as adaptation mechanisms attenuate the phasic signal within the first few seconds of stimulation. Consistent with this hypothesis, studies using shorter trial durations have successfully demonstrated speed-dependent effects on postural sway [25,26], and the known latency of optic-flow perception has been estimated at 4 to 7 s after stimulus onset [27], suggesting that the early temporal window preceding conscious perception may be particularly informative about the direct sensorimotor coupling between optic flow and postural muscle activation.

Despite the theoretical importance of early postural responses, no study has systematically characterised the time-domain structure of postural muscle activation in response to optic-flow stimuli varying in speed and retinal location. Specifically, it remains unknown whether optic-flow velocity and retinal stimulation location modulate neuromuscular responses differently within the first milliseconds following stimulus onset. This distinction would have important implications for understanding the neural mechanisms underlying visually driven postural control.

This study aimed to determine whether optic-flow velocity and retinal stimulation location modulate early postural muscle activity and whether these effects differ between two consecutive temporal windows following stimulus onset (0–200 ms and 200–400 ms). To address this aim, we reanalysed a previously published EMG dataset collected from healthy adults [14]. We examined four muscles, the tibialis anterior, soleus, trapezius descendens and paraspinal C4, which were selected to capture the full rostrocaudal extent of the postural response, from distal lower-limb stabilisers to proximal trunk and cervical muscles. By isolating the phasic component of the neuromuscular response during the earliest stages of visually evoked postural processing [27,28], this approach tests the hypothesis that optic-flow velocity and retinal location exert a significant and time-dependent modulation of postural muscle activation that is not detectable in analyses based on mean activity over long trial durations.

## 2. Materials and Methods

### 2.1. Participants

Twenty healthy volunteers (10 females, 10 males) participated in this study. The participants were the same cohort described in Raffi et al. [14]. With respect to the previous paper, two participants were excluded from the final analysis due to missing recordings required as covariates in the statistical model (see Section 2.5). Thus, the final sample comprised 18 participants (10 females, 8 males) with a mean age of 28.5 ± 4.05 years and mean BMI of 21.83 ± 2.33 kg/m^2^. None of the participants were taking medications or supplements, and none reported physical deficits or muscular injuries at the time of the study. All subjects had normal or corrected-to-normal vision.

Hand and foot dominance were used to characterise the study sample and were assessed using the Waterloo Footedness Questionnaire (WFQ) and Waterloo Handedness Questionnaire (WHQ) [29,30]. For each questionnaire, a laterality index was calculated according to the following formula:

[(right preference − left preference)/(right preference + left preference)] × 100

A positive laterality index indicates a right dominance, while a negative index a left dominance.

Written informed consent was obtained from all participants prior to data collection. The experimental protocol was approved by the Bioethics Committee of the University of Bologna (protocol code 224109, approval date 26 September 2019) and conducted in accordance with the Declaration of Helsinki.

### 2.2. Optic-Flow Stimuli

**Figure 1:**
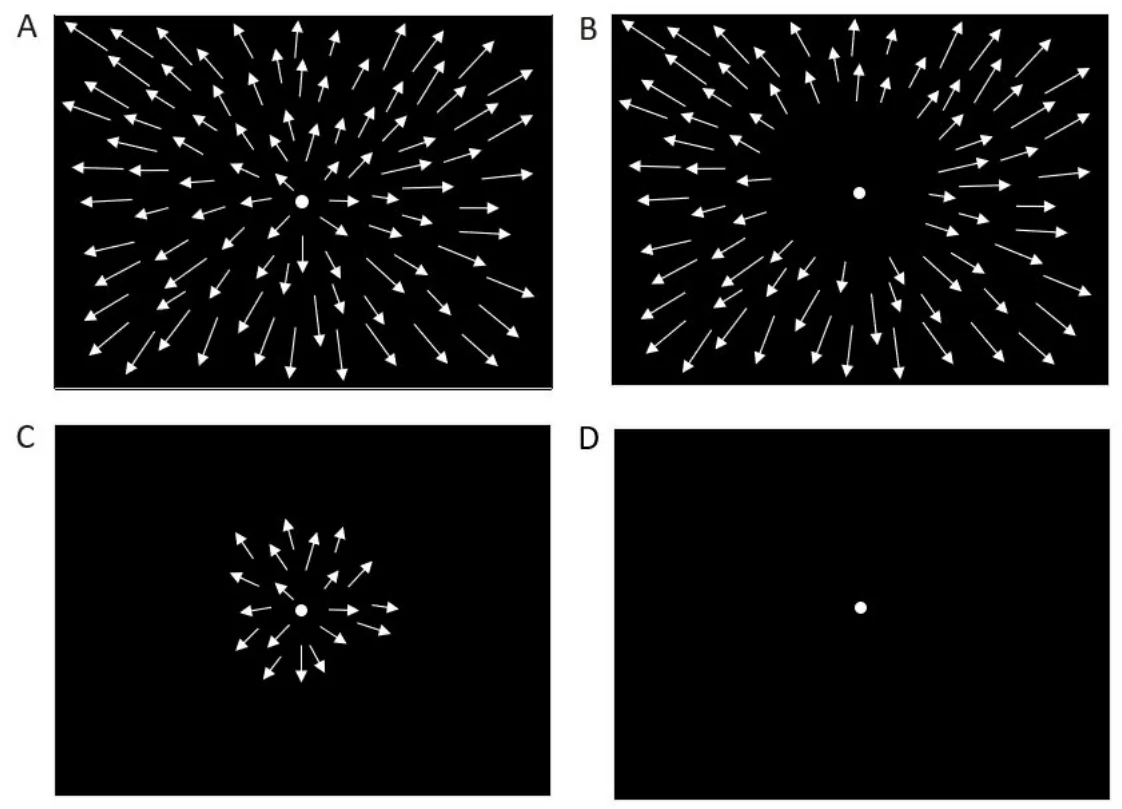
Radial optic-flow stimuli. (**A**). Large-field stimulus, in which white dots moved radially from the centre to the periphery across the entire visual field (135 × 107°). (**B**). Peripheral stimulus, restricted to the peripheral visual field; the black area in the center has a radius of 20°. (**C**). Foveal stimulus, restricted to the central visual field, with a radius of 7°. (**D**). Baseline condition: dark screen with a central fixation point and no optic flow. Arrows represent the velocity vectors of the moving dots, increasing in magnitude from centre to periphery. Stimuli were presented at five speeds: 8, 11, 14, 17 and 20°/s. The fixation point was displayed in all conditions. Schematic representation, not to scale.

Radial optic-flow stimuli were presented as described in detail in Raffi et al. [14]. Briefly, visual stimuli consisted of white dots (luminance 1.3 cd/m^2^, size 0.4°) back-projected onto a translucent screen subtending 135 × 107° of the visual field. Stimuli were presented in three retinal configurations: large-field, peripheral and foveal (Figure 1). These stimulus configurations were designed to vary the spatial extent of optic-flow stimulation across the visual field. The subjects were positioned 115 cm away from the screen. A fixation point was always displayed at the centre of the screen. Participants were instructed to maintain fixation and not to resist the optic-flow stimulus. Each radial expanding optic flow was presented at five speeds (8, 11, 14, 17 and 20°/s, Figure 1A–C), which refer to the nominal dot-speed parameter used to scale the radial expansion field. To simulate radial optic flow, local dot velocity increased with eccentricity from the focus of expansion. A dark-fixation baseline (no optic flow) served as the control condition (Figure 1D). Each condition was presented twice, for a total of 32 trials per participant. Trial duration was 30 s. The stimuli were randomly presented to prevent selection bias. The randomization was identical for all subjects to create a homogeneous treatment between participants, without involving any potential biases or judgments. Stimuli were generated using the user-contributed MATLAB Psychophysics Toolbox (The MathWorks Inc., Natick, MA, USA).

### 2.3. Electromyographic Recordings

Surface EMG signals were recorded bilaterally from four muscles: the tibialis anterior (TA), soleus (SOL), trapezius descendens (TD) and cervical paraspinals at C4. These muscles were selected to sample different contributions to lower-limb and cervical/shoulder postural control while allowing assessment of left–right differences. Recordings were performed using disposable Ag/AgCl electrodes (32 × 32 mm) in a bipolar configuration, positioned over the muscle bellies (RAM Apparecchi Medicali srl, Genova, Italy).

EMG signals were acquired at 1000 Hz using a FREE1000 EMG system (BTS Bioengineering Inc., Garbagnate Milanese, Italy). The eight-channel recording system limited simultaneous bilateral acquisition to four muscles. Prior to the experimental session, maximum voluntary contraction (MVC) was recorded for each muscle using isometric dynamometry. All EMG signals were subsequently normalised to the individual MVC peak to allow comparisons across muscles and participants [14].

### 2.4. EMG Signal Processing

EMG signals were band-pass filtered (Butterworth, 20–450 Hz) and positively rectified using SMART Analyzer software (BTS Bioengineering Inc., Garbagnate Milanese, Italy). Normalised root mean square (RMS) values were computed in 100 ms bins from the onset of each stimulus using MATLAB version 19 (The Mathworks Inc., Natick, MA, USA).

Trials were excluded when recording artefacts compromised the quality of the EMG signals. A total of four trials were rejected: two in the baseline condition and two in the foveal stimulation condition in four different subjects.

To isolate the phasic neuromuscular response before motor adaptation mechanisms are expected to influence the neural response, the present analysis focused on the first 400 ms following stimulus onset, partitioned into two consecutive temporal windows: an early window (0–200 ms) and a late window (200–400 ms). The choice of two 200 ms windows was informed by converging physiological considerations. The early window (0–200 ms) is expected to be dominated by short-latency, largely reflexive contributions: visual motion processing engages occipital and extrastriate cortical areas within the first 200 ms after stimulus onset [31], while early vestibulo-ocular responses have also been reported within a comparable temporal range [32]. The later window (200–400 ms) was therefore expected to capture the onset of longer-latency contributions, including early corticospinal integration of visual and postural information, consistent with visually evoked postural responses emerging within the first few hundred milliseconds following stimulus onset and preceding conscious perception of self-motion [27,28]. It should be noted that these temporal windows precede the longer integration times required for conscious optic-flow perception and vection [33]. Because the underlying EMG signal was quantified in 100 ms bins (see above), the 200 ms partition represents an aggregation of two such bins per window, chosen to balance temporal specificity against the signal stability required for reliable RMS estimation; narrower windows increased trial-to-trial variability in pilot inspection of the binned data, while wider windows would have merged the short- and longer-latency components the analysis was designed to distinguish.

### 2.5. Statistical Analysis

A repeated measures ANCOVA was performed for each muscle (TA, SOL, TD, C4). The within-subject factors were: time (0–200 ms, 200–400 ms), velocity (8, 11, 14, 17, 20°/s), stimuli (large-field, foveal, peripheral) and laterality (left, right). Sex (male, female) was included as a between-subjects factor.

Baseline RMS values, computed during the dark-fixation condition in the corresponding temporal windows, were included as covariates to account for interindividual differences in spontaneous muscle activity unrelated to optic-flow stimulation. Four baseline covariates were entered for each muscle, corresponding to the combinations of laterality and time. Before analysis, each baseline covariate was mean-centred using the mean of the participants with complete data for the corresponding muscle. Consequently, within-subject effects were evaluated at the mean baseline level of the analysed sample, consistent with the reference values used to calculate the estimated marginal means (EMMs).

Estimated marginal means were calculated with equal weighting of the two Sex groups. Greenhouse–Geisser corrections were applied to all within-subject effects involving factors with more than one numerator degree of freedom. Effects involving two-level factors did not require a sphericity correction. Effect sizes are reported as partial eta squared (η^2^p), and statistical significance was set to *p* < 0.05.

Where appropriate, pairwise comparisons among the five velocity levels were performed using Bonferroni adjustment for the ten possible pairwise comparisons. In addition, linear and quadratic polynomial contrasts across the five equally spaced velocity levels were examined. Global quadratic contrasts were adjusted using Holm’s procedure across the four muscles. For configuration-specific analyses, linear and quadratic contrasts were evaluated separately for each of the three retinal stimulation conditions, with Holm correction applied to the six contrasts within each muscle.

All statistical analyses were performed using IBM SPSS Statistics, version 29.0 (IBM Corp., Armonk, NY, USA).

## 3. Results

The analysis of the WFQs and WHQs showed that sixteen participants were strongly right-handed and right-footed, whereas two participants showed left dominance.

A significant main effect of time was observed in all four muscles, with higher adjusted EMG activity during the 200–400 ms post-stimulus window.

### 3.1. Tibialis Anterior (TA)

For the tibialis anterior, a significant main effect of time was observed (F(1, 12) = 14.60, *p* = 0.002, η^2^p = 0.549), with adjusted EMG activity increasing from 1.317% MVC during the 0–200 ms window to 1.490% MVC during the 200–400 ms window. The main effect of velocity approached, but did not reach, the conventional significance threshold after Greenhouse–Geisser correction (F(2.67, 32.08) = 3.00, *p* = 0.050, η^2^p = 0.200). Neither the main effect of stimuli (F(1.28, 15.41) = 2.72, *p* = 0.113, η^2^p = 0.185) nor laterality (F(1, 12) = 1.75, *p* = 0.211, η^2^p = 0.127) was significant.

A significant velocity × laterality interaction was identified (F(2.79, 33.46) = 3.52, *p* = 0.028, η^2^p = 0.227). However, follow-up comparisons did not identify a significant right–left difference at any individual velocity after Holm correction, and neither the linear nor quadratic velocity trends differed significantly between sides after correction.

**Figure 2:**
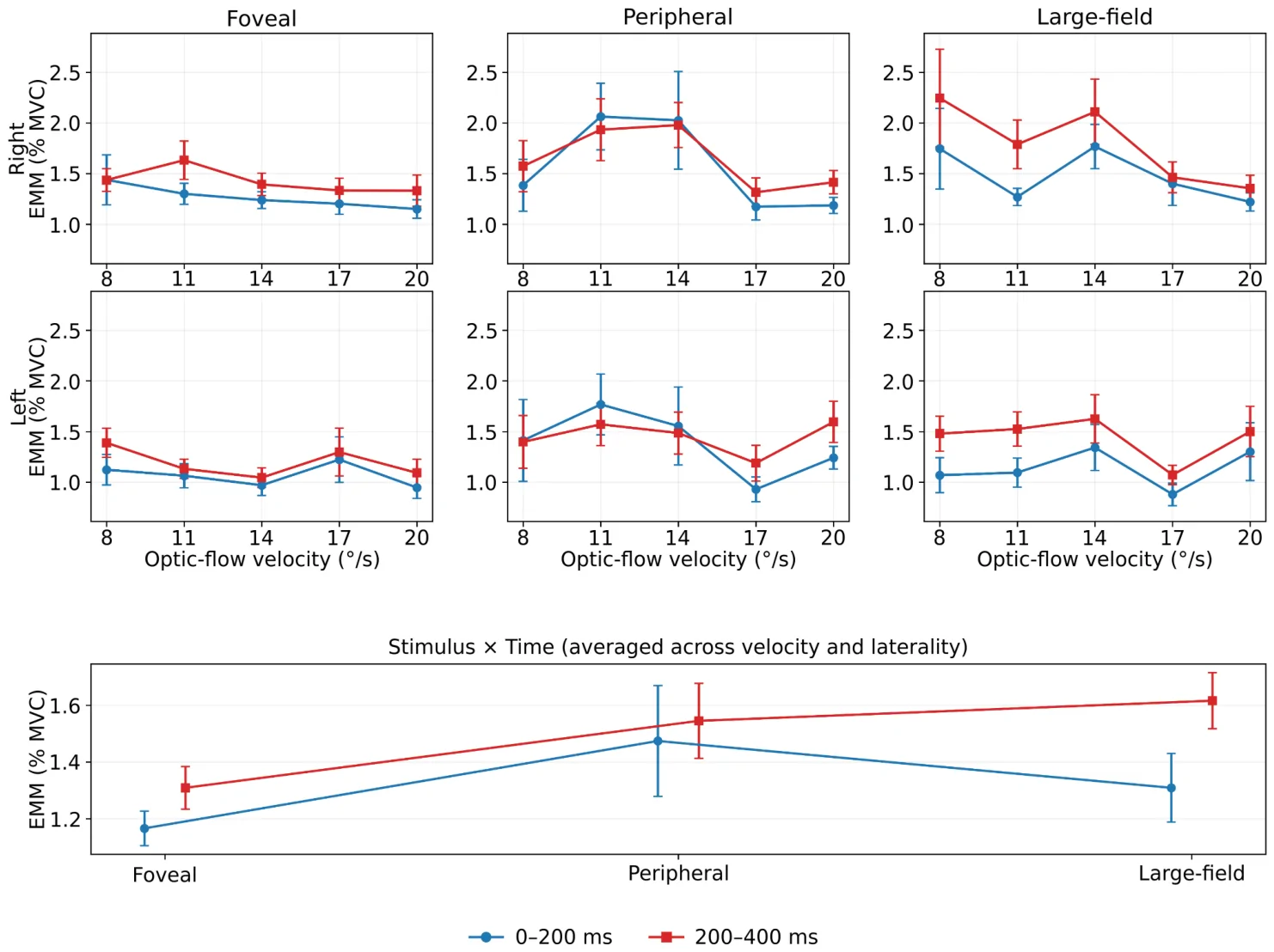
Time course of tibialis anterior (TA) muscle activation during optic-flow stimulation. Estimated marginal means (EMM ± SE, % MVC) are shown separately for the (**right**) and (**left**) TA muscles during the 0–200 ms and 200–400 ms post-stimulus windows, as a function of optic-flow velocity (8, 11, 14, 17, and 20°/s) and retinal stimulation location (foveal, peripheral, and large-field). The (**lower panel**) shows the stimuli × time effect averaged across velocity and laterality. The temporal increase was significantly greater during large-field than during peripheral stimulation (Holm-adjusted *p* = 0.026). EMMs are adjusted for baseline RMS activity and sex.

A significant stimuli × time interaction was observed (F(1.93, 23.19) = 4.24, *p* = 0.028, η^2^p = 0.261). The temporal increase in activation was greater during large-field than during peripheral stimulation, with a difference in temporal change of 0.236 percentage points MVC (Holm-adjusted *p* = 0.026). Within individual stimulus conditions, activation increased significantly over time during foveal and large-field stimulation, whereas the increase during peripheral stimulation was not significant after correction (Figure 2).

Polynomial analyses did not identify a significant quadratic velocity trend when averaged across stimulus conditions, laterality and time. Within the peripheral stimulation condition, however, a significant negative quadratic component was observed (F(1, 12) = 13.17, nominal *p* = 0.003; Holm-adjusted *p* = 0.021 across the six within-TA linear and quadratic contrasts). This finding did not remain significant when correction was extended across the corresponding contrasts from all four muscles.

### 3.2. Soleus (SOL)

For the soleus, a significant main effect of time was observed (F(1, 11) = 71.27, *p* < 0.001, η^2^p = 0.866). Adjusted EMG activity increased from 4.867% MVC during the 0–200 ms window to 5.499% MVC during the 200–400 ms window. No significant main effects of velocity (F(3.21, 35.32) = 1.11, *p* = 0.362, η^2^p = 0.091), laterality (F(1, 11) = 0.12, *p* = 0.733, η^2^p = 0.011), or stimuli (F(1.59, 17.46) = 2.74, *p* = 0.102, η^2^p = 0.199) were identified.

A significant velocity × laterality × time interaction was observed (F(3.48, 38.29) = 3.43, *p* = 0.021, η^2^p = 0.238). Follow-up analysis showed that at 8°/s, averaged across retinal stimulation configurations, the increase from the 0–200 ms to the 200–400 ms window was 0.420 percentage points of MVC greater on the right than on the left side (Holm-adjusted *p* = 0.040 across the five velocities).

**Figure 3:**
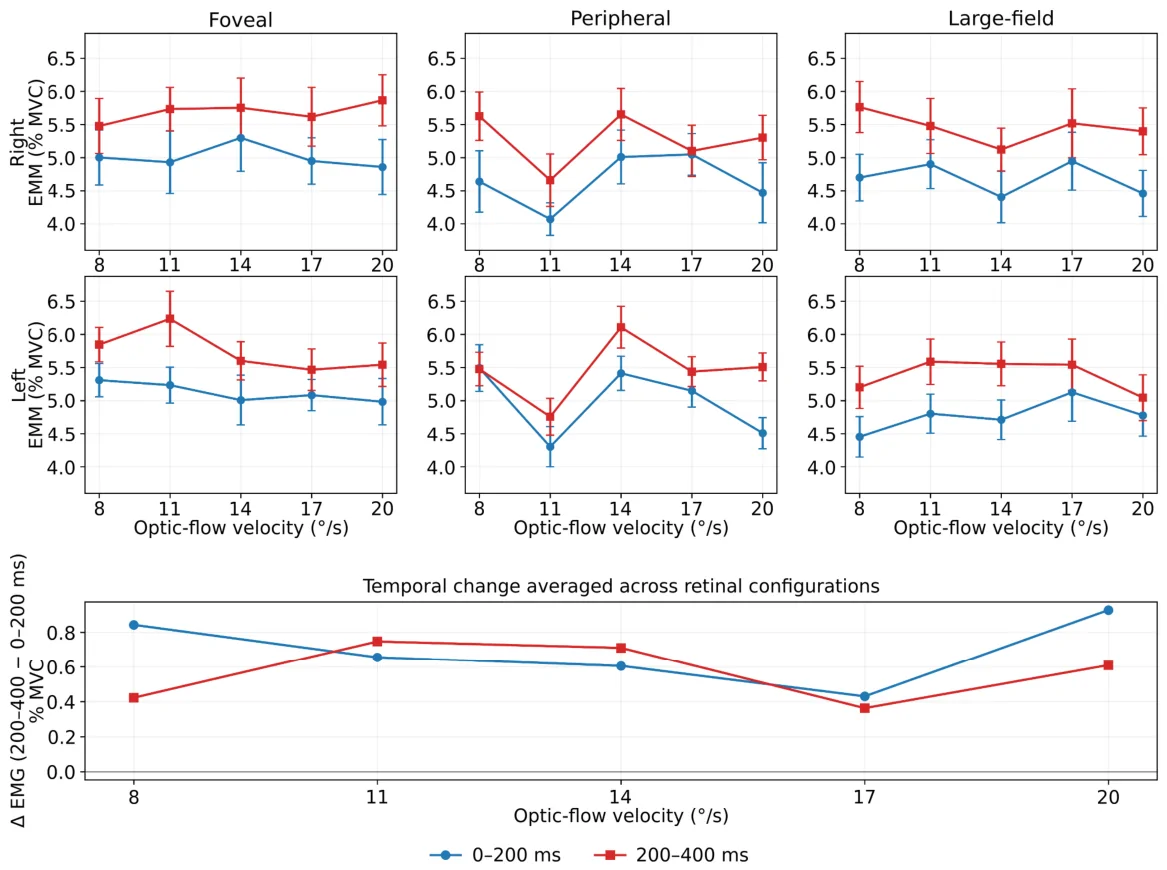
Time course of soleus (SOL) muscle activation during optic-flow stimulation. Estimated marginal means (EMM ± SE, % MVC) are shown separately for the (**right**) and (**left**) SOL muscles during the 0–200 ms and 200–400 ms post-stimulus windows, as a function of optic-flow velocity (8, 11, 14, 17, and 20°/s) and retinal stimulation location (foveal, peripheral, and large-field). The (**lower panel**) shows the temporal change in activation (Δ = 200–400 ms minus 0–200 ms), averaged across retinal stimulation configurations, for the (**right**) and (**left**) sides. At 8°/s, the temporal increase was significantly greater on the (**right**) than on the (**left**) side (Holm-adjusted *p* = 0.040). EMMs are adjusted for baseline RMS activity and sex.

A significant velocity × laterality × stimuli × time interaction was also observed (F(3.62, 39.85) = 2.79, *p* = 0.044, η^2^p = 0.202), indicating that the temporal pattern depended jointly on velocity, body side and retinal stimulation configuration (Figure 3).

No other interaction among the experimental within-subject factors reached statistical significance after Greenhouse–Geisser correction.

### 3.3. Trapezius Descendens (TD)

For the trapezius descendens, a significant main effect of time was found (F(1, 12) = 25.95, *p* < 0.001, η^2^p = 0.684). Adjusted EMG activity increased from 0.789% MVC in the 0–200 ms window to 0.913% MVC in the 200–400 ms window.

**Figure 4:**
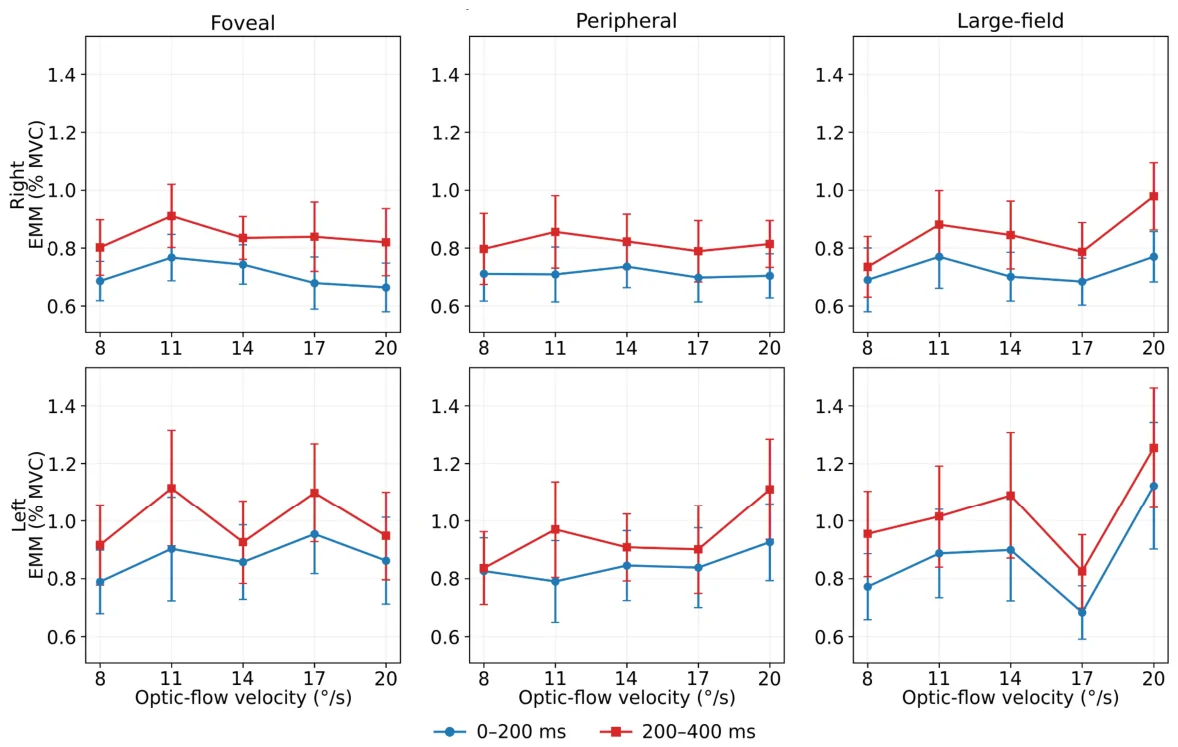
Time course of trapezius descendens (TD) muscle activation during optic-flow stimulation. Estimated marginal means (EMM ± SE, % MVC) are shown separately for the (**right**) and (**left**) TD muscles during the 0–200 ms and 200–400 ms post-stimulus windows, as a function of optic-flow velocity (8, 11, 14, 17, and 20°/s) and retinal stimulation location (foveal, peripheral, and large-field). EMMs are adjusted for baseline RMS activity and sex.

A significant main effect of velocity was also observed (F(2.59, 31.06) = 3.53, *p* = 0.031, η^2^p = 0.227). However, none of the ten pairwise comparisons between individual velocity levels remained significant after Bonferroni correction. Similarly, a linear contrast across velocity levels was nominally significant (*p* = 0.022) but did not remain significant after Holm adjustment across the global velocity-trend analyses. Therefore, the velocity effect does not identify a specific preferred velocity or establish a robust monotonic relationship between optic-flow speed and TD activation (Figure 4).

Neither laterality (F(1, 12) = 1.07, *p* = 0.321, η^2^p = 0.082) nor stimuli (F(1.29, 15.46) = 0.58, *p* = 0.501, η^2^p = 0.046) showed significant main effects. No interaction among velocity, laterality, stimuli and time reached statistical significance after Greenhouse–Geisser correction.

### 3.4. Paraspinal C4 (C4)

For the paraspinal C4 muscle, a significant main effect of time was observed (F(1, 12) = 28.70, *p* < 0.001, η^2^p = 0.705). Adjusted EMG activity increased from 3.817% MVC during the 0–200 ms window to 4.095% MVC during the 200–400 ms window.

**Figure 5:**
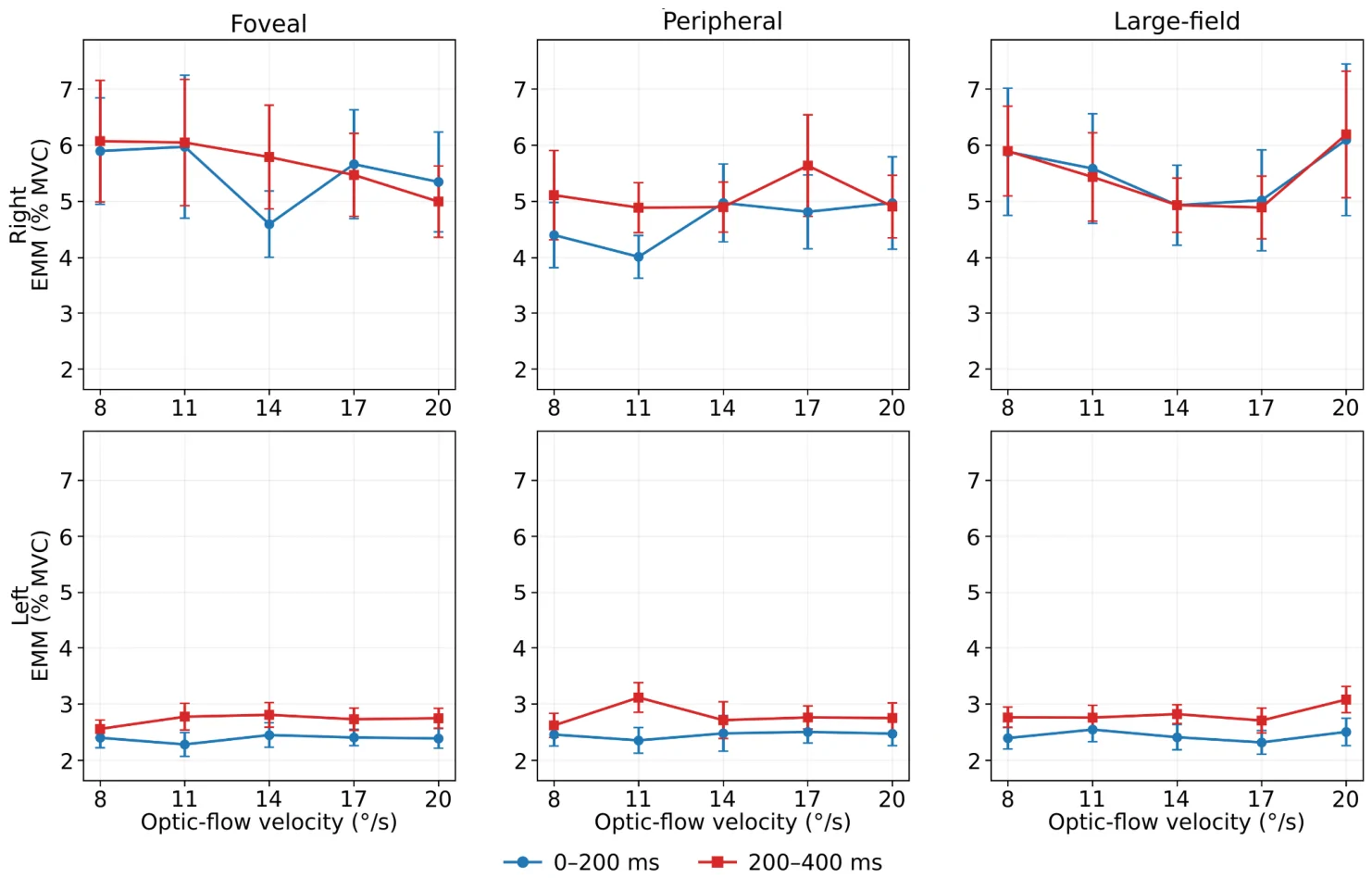
Time course of paraspinal C4 muscle activation during optic-flow stimulation. Estimated marginal means (EMM ± SE, % MVC) are shown separately for the (**right**) and (**left**) C4 muscles during the 0–200 ms and 200–400 ms post-stimulus windows, as a function of optic-flow velocity (8, 11, 14, 17, and 20°/s) and retinal stimulation location (foveal, peripheral, and large-field). EMMs are adjusted for baseline RMS activity and sex.

A significant main effect of laterality was also found (F(1, 12) = 15.19, *p* = 0.002, η^2^p = 0.559), indicating different activation levels between the right and left C4 muscles. In addition, a significant laterality × stimuli interaction was observed (F(1.14, 13.65) = 4.59, *p* = 0.047, η^2^p = 0.277). Follow-up comparisons showed greater right than left C4 activation in all three retinal stimulation configurations after Holm correction. However, the magnitude of the right–left difference did not differ significantly between any specific pair of stimulus configurations after correction (Figure 5).

The main effect of velocity was not significant (F(2.17, 26.05) = 1.59, *p* = 0.222, η^2^p = 0.117). The main effect of stimuli also did not reach statistical significance (F(1.18, 14.14) = 3.98, *p* = 0.061, η^2^p = 0.249). The velocity × stimuli × time interaction approached but did not reach significance (F(1.98, 23.76) = 3.38, *p* = 0.052, η^2^p = 0.220), while the velocity × laterality × stimuli × time interaction was not significant (F(1.72, 20.65) = 2.83, *p* = 0.089, η^2^p = 0.191). Likewise, the velocity × laterality × stimuli interaction did not remain significant after correction (F(1.62, 19.47) = 3.04, *p* = 0.080, η^2^p = 0.202). Thus, the principal C4 findings were a marked temporal increase in activation and a clear right–left asymmetry.

A summary of all significant effects across muscles is presented in Table 1.

## 4. Discussion

The present study investigated the temporal organisation of postural muscle activity during the first 400 ms following optic-flow onset, with particular attention to the effects of stimulus velocity, retinal stimulation location, body side, and their interaction with time. The main finding of the reanalysis is a consistent temporal evolution of EMG activity across all four muscles examined. All muscles showed greater activation during the 200–400 ms post-stimulus window than during the 0–200 ms post-stimulus window. Further, more selective muscle-specific effects involving velocity, retinal stimulation configuration, and laterality were observed.

This revised pattern of findings provides a different perspective from analyses based on mean EMG activity over prolonged stimulation periods. The present results suggest that the early post-stimulus period is characterised by a distributed temporal adjustment involving distal, antigravity, proximal, and cervical muscles. At the same time, the influence of optic-flow characteristics was not uniform across the postural musculature, indicating that the temporal evolution of the response and its stimulus-dependent modulation should be considered as partially distinct components.

### 4.1. A Distributed Temporal Response Across Postural Muscles

The observation of a significant time effect in all four muscles indicates that postural muscle activity is not stationary during the first 400 ms following stimulus onset. The magnitude of this temporal effect was particularly pronounced in SOL and C4, but was also present in TA and TD. This common increase suggests that the transition from the first to the second 200 ms interval involves a coordinated adjustment across different levels of the postural system. Such a distributed pattern is compatible with the organisation of postural control as a multisegmental process. Distal lower-limb muscles contribute to ankle-based postural stabilisation, whereas proximal and cervical muscles contribute to upper-body and head stabilisation and to the coordination of head and gaze [34,35,36]. The presence of temporal modulation at several rostrocaudal levels therefore suggests that the early post-stimulus period involves a broad reorganisation of postural muscle activity. However, the present data do not establish the mechanism responsible for this increase. The temporal change may include visually driven postural adjustments, general responses to stimulus onset, or other nonspecific components. In particular, a startle-like or orienting response cannot be completely excluded from the present experimental design. Simultaneous analysis of EMG, centre-of-pressure displacement, and body kinematics will be required to determine how the observed muscle activation relates to actual postural behaviour.

### 4.2. Muscle-Specific Modulation by Velocity, Retinal Stimulation, and Laterality

Although time was the most consistent effect across muscles, additional effects differed substantially between muscle groups.

In TA, the results indicated that the influence of velocity was not identical on the two sides. However, no individual right–left comparison at a specific velocity remained significant after correction, preventing attribution of this interaction to a particular optic-flow speed. The temporal increase was greater during large-field than during peripheral stimulation, suggesting that the spatial distribution of visual motion can influence the temporal development of distal lower-limb activation. The results of the quadratic analysis suggest a non-linear relationship between velocity and TA activation under this specific visual configuration. The result therefore provides a hypothesis for future investigation.

SOL is a primary antigravity muscle that maintains substantial tonic activity during quiet standing [35,36]. Despite this tonic background, SOL showed the largest temporal effect among the muscles examined, with a marked increase in activity between the 0–200 ms and 200–400 ms windows. This temporal modulation also depended on optic-flow velocity and body side: at 8°/s, the increase between the two temporal windows was greater on the right than on the left when averaged across retinal stimulation conditions. The results further suggest that this temporal pattern was influenced by retinal stimulation configuration. However, given the complexity of the four-way interaction and the limited sample size, it would be inappropriate to attribute the effect to a specific combination of velocity, side, and retinal location. The absence of significant main effects of velocity or stimuli further indicates that the SOL response cannot be described as a simple increase or decrease according to optic-flow speed or retinal location. Stimulus-related effects emerged through their interaction with time and laterality. These findings suggest that tonic background activation does not preclude rapid modulation of SOL activity during the early post-stimulus period and reinforce the importance of analysing the temporal structure of postural EMG.

TD showed both a significant temporal increase and a significant effect of velocity that seem to vary across velocity conditions, but the present data do not support either a preferred velocity or a clear monotonic velocity–response relationship. The temporal increase may reflect an early contribution of upper-body stabilisation, consistent with the role of the trapezius in shoulder-girdle and postural control [35], although the functional significance of this activation cannot be determined from EMG alone.

### 4.3. Cervical Paraspinal Activity and Lateral Asymmetry

The principal C4 findings were a pronounced effect of time and a clear effect of laterality. Cervical paraspinal activation was greater on the right than on the left, and this lateral difference was observed across all three retinal stimulation conditions. The magnitude of the asymmetry varied across visual stimuli, although follow-up comparisons did not identify a specific pair of configurations that differed significantly in the size of the right–left effect.

The presence of lateralised cervical activation is physiologically interesting because cervical muscles contribute to head stabilisation and gaze control during self-motion and interact with vestibular and visual signals through cervico-ocular and vestibulo-cervical mechanisms [34,37]. Nevertheless, the present right–left difference should not be interpreted as evidence of a direct relationship with handedness or footedness. Although most participants were right-dominant, the study was not designed or sufficiently powered to test whether behavioural dominance explains cervical asymmetry.

The relatively large variability in right C4 activation also warrants caution. The laterality effect was statistically robust, but the magnitude and functional meaning of this asymmetry should be confirmed in larger samples and with additional kinematic measures of head and trunk behaviour.

### 4.4. Methodological Implications of an Early Time-Domain Approach

A central contribution of the present study is the demonstration that dividing the early post-stimulus period into consecutive temporal windows reveals changes in muscle activity that are not apparent when EMG is averaged across the entire 30 s stimulation period. In our previous work [14], the absence of clear stimulus-dependent effects on mean EMG activity was interpreted as potentially reflecting adaptation during prolonged visual stimulation. The present results are compatible with the existence of early neuromuscular dynamics that become diluted when activity is averaged over long intervals. However, the present analysis cannot establish that adaptation is responsible for the difference between early and whole-trial results. Only the first 400 ms were analysed here, and no direct comparison was made with later portions of the same trial. The difference between the two analytical approaches could therefore reflect adaptation, temporal averaging of a brief onset response, or a combination of both mechanisms. Future studies should examine EMG continuously from stimulus onset throughout the entire period of optic-flow exposure. Such analyses would allow determination of whether the early response subsequently decreases, remains sustained, or evolves into a different pattern of muscle recruitment. A larger number of repeated trials would also make it possible to examine whether the magnitude of the early response changes across successive stimulus presentations, which could provide evidence for habituation, predictive attenuation, or adaptation.

The current results also show the importance of considering laterality. Averaging left and right muscles can conceal effects that depend on body side, as illustrated particularly by SOL and C4. Future studies of visually induced postural responses should therefore consider bilateral recordings not only as replicate measurements but as potentially informative components of the sensorimotor response.

From a broader perspective, time-resolved EMG analysis may provide a useful framework for investigating alterations of visually driven postural control in ageing and clinical populations.

### 4.5. Limitations

Several limitations should be acknowledged. First, the relatively small sample size limits the interpretation of complex higher-order interactions and reduces the generalisability of the findings.

Second, the original protocol was not designed for a specifically temporal analysis and each condition was repeated only twice. The present time-window analysis was therefore retrospective and may be affected by trial-to-trial variability.

Third, dark-fixation baseline activity was included as a covariate. Accordingly, the significant time effects describe differences between the two post-stimulus windows and do not constitute a direct comparison between optic-flow stimuli and baseline activity.

Finally, the study included only four bilateral muscle groups in healthy young adults. Future studies with larger samples, more repetitions, and simultaneous EMG, kinematic and postural measurements are needed to confirm the present findings and clarify their functional significance.

## 5. Conclusions

The present study shows that the earliest postural response to optic flow is characterised by a rapid temporal reorganisation of muscle activity across different levels of the postural system. Although this temporal increase was common to all muscles examined, the influence of optic-flow velocity, retinal stimulation and laterality was muscle-specific, revealing a differentiated rather than uniform neuromuscular response.

These findings indicate that analysing EMG within short post-stimulus windows can uncover early visuomotor dynamics that are lost when activity is averaged over prolonged stimulation periods. Time-resolved bilateral EMG therefore provides a useful approach for investigating how visual motion information is transformed into postural motor activity.

## Figures and Tables

**Table 1:** Summary of statistically significant within-subject ANCOVA effects for each postural muscle examined. For each significant effect, the *p*-value and partial eta squared (η^2^p) are reported. Effects that did not reach statistical significance are indicated as ns (*p* > 0.05). Greenhouse–Geisser corrections were applied to within-subject effects involving factors with more than one numerator degree of freedom. TA = tibialis anterior; SOL = soleus; TD = trapezius descendens; C4 = paraspinal C4.

Effect	TA	SOL	TD	C4
Velocity	ns	ns	*p* = 0.031, η^2^p = 0.227	ns
Laterality	ns	ns	ns	*p* = 0.002, η^2^p = 0.559
Time	*p* = 0.002, η^2^p = 0.549	*p* < 0.001, η^2^p = 0.866	*p* < 0.001, η^2^p = 0.684	*p* < 0.001, η^2^p = 0.705
Velocity × Laterality	*p* = 0.028, η^2^p = 0.227	ns	ns	ns
Stimuli × Time	*p* = 0.028, η^2^p = 0.261	ns	ns	ns
Laterality × Stimuli	ns	ns	ns	*p* = 0.047, η^2^p = 0.277
Velocity × Laterality × Time	ns	*p* = 0.021, η^2^p = 0.238	ns	ns
Velocity × Laterality × Stimuli × Time	ns	*p* = 0.044, η^2^p = 0.202	ns	ns

## Data Availability

The data that support the findings of this study are available from the corresponding author upon reasonable request due to privacy regulations and institutional data governance policies.

## Abbreviations

The following abbreviations are used in this manuscript:

TA	Tibialis Anterior
SOL	Soleus
TD	Trapezius Descendens
C4	Paraspinal C4
EMG	Electromyography
